# Pathways to enhancing prenatal diagnosis of skeletal dysplasias

**DOI:** 10.1002/pmf2.70302

**Published:** 2026-05-04

**Authors:** Kate Swanson, Mary E. Norton

**Affiliations:** ^1^ Department of Obstetrics, Gynecology, and Reproductive Sciences Division of Maternal‐Fetal Medicine University of California San Francisco California USA; ^2^ Department of Pediatrics Division of Medical Genetics University of California San Francisco California USA; ^3^ Department of Obstetrics & Gynecology Division of Maternal‐Fetal Medicine Intermountain Healthcare Salt Lake City Utah USA; ^4^ Center for Maternal‐Fetal Precision Medicine University of California San Francisco California USA; ^5^ Institute for Human Genetics University of California San Francisco California USA

Skeletal dysplasias are a group of congenital disorders of bone and cartilage growth that lead to abnormal development of the skeleton. Clinically, this can result in disproportionately short stature, altered bone shape and structure, and neurological or respiratory complications. Over 750 skeletal dysplasias have been described, with many overlapping features. Historically, their classification has relied on phenotypic features identified largely by imaging and radiographic findings. More recently, with advances in genetic testing and broader use of genomic sequencing, the molecular basis of many of these disorders has been described and this has become an important component of their classification.

In this issue of “*Pregnancy*,” Wang et al. present a scoping review of the prenatal diagnosis of skeletal dysplasias, including a review of embryology, diagnostic imaging features associated with different conditions, and considerations in predicting lethality when a skeletal dysplasia is suspected [1]. The authors also review the genetic basis for some of the more common conditions and argue that given the extensive breadth of genetic etiologies, exome or genome sequencing should be considered first‐line testing when a skeletal dysplasia is prenatally suspected. Their review can serve as a guide for clinicians evaluating shortened long bones and other features suggestive of skeletal dysplasia prenatally.

Making a prenatal diagnosis of a specific skeletal dysplasia is more than an esoteric exercise; it can inform reproductive decision‐making, delivery planning, care direction at the time of delivery, and neonatal surveillance. Additionally, a diagnosis informs long‐term management and recurrence risk. Increasingly, treatments are available for skeletal dysplasias, though in some cases, their use remains experimental or controversial [2].

While genomic analysis with exome or genome sequencing has transformed the evaluation and diagnosis of many rare diseases, including skeletal dysplasias, it does not replace detailed phenotyping. Rather, making a genetic diagnosis is an iterative process in which a phenotype is identified and sequencing performed; the suspected variants then inform deeper phenotyping to confirm that the variants fit with the clinical features. Such deep phenotyping can help to confirm a diagnosis or expand the phenotype associated with a given condition. The process is complex, and it is important for clinicians to appreciate that while laboratories identify variants, clinicians make diagnoses by applying the identified variants to a specific phenotype. Wang et al. apply this process to the diagnosis of skeletal dysplasias; appreciating the importance of detecting skeletal dysplasias prenatally, they describe the ultrasound features that are detectable as early as the first trimester and should prompt a thorough evaluation for underlying skeletal dysplasia. They outline comprehensive imaging that should be performed to better refine diagnosis and prognosis, recommend exome or genome sequencing as first‐line diagnostic testing to identify a specific diagnosis when possible, and suggest adjunctive imaging strategies such as 3‐D ultrasound, magnetic resonance imaging (MRI), and computed tomography (CT) scan in some situations.

While their recommendations are comprehensive and may offer the most up‐to‐date information to guide families, it is important to acknowledge that these tools may not be accessible to all patients. Current guidance from both the Society for Maternal‐Fetal Medicine and the International Society for Prenatal Diagnosis includes consideration of exome or genome sequencing when a single‐gene disorder (such as skeletal dysplasia) is suspected that is unlikely to be identified on chromosomal microarray. However, there is no significant discussion of when sequencing should be considered rather than a gene panel [3, 4]. Evidence suggests that payors still cover gene panels preferentially over exome and genome sequencing in most cases [5]. And while exome and genome have excellent detection rates for genetic etiologies of skeletal dysplasias, gene panels for this phenotype offer improved coverage compared to other prenatal anomalies [6]. Similarly, fetal MRI and CT may offer significant advantages over ultrasound alone, but patients may need to travel a significant distance to be seen by specialized providers and insurers may not cover this additional imaging. While comprehensive genetic and imaging evaluation in the setting of prenatally suspected skeletal dysplasia is key to improving diagnosis and outcomes, more work on improving equitable access to this testing is needed.

In addition to the overlapping features of many skeletal dysplasias, another limitation of prenatal diagnosis of these disorders is that some do not typically present until the third trimester, when not all patients routinely undergo ultrasound surveillance. While these are less likely to be lethal anomalies, their prenatal diagnosis can still significantly impact management during pregnancy and the newborn period. For example, when achondroplasia is diagnosed, ultrasound monitoring of head size is important to inform delivery planning (as cesarean may be recommended in the setting of macrocephaly), and delivery should occur at an institution with pediatric providers who can assess the craniocervical junction shortly after delivery [7]. The authors mention single‐gene non‐invasive prenatal diagnostic testing (sgNIPT), which may identify de novo or paternally inherited pathogenic or likely pathogenic variants in FGFR2, FGFR3, COL1A1, and COL1A2. However, data on the positive predictive value in a low‐risk population are lacking, and at this time, current guidelines do not recommend routine screening with sgNIPT. Nevertheless, these noninvasive options may be useful, and more acceptable to patients and providers than diagnostic testing with amniocentesis when a nonspecific finding such as shortened long bones is detected in the third trimester.

As the authors mention, even when a genetic diagnosis is made, there can be significant variation in expressivity, making it difficult to truly predict outcomes and complicating parents' ability to make decisions. This is likely where enhanced prenatal phenotyping with adjunctive imaging techniques has an important role, even in the era of genome sequencing, and this will only increase in the future. As we improve our prenatal phenotyping and correlate findings with genetic results and long‐term outcomes, we will continue to improve our ability to prognosticate when a prenatal diagnosis is made.
